# Dr. Merriman on Mandrakes

**Published:** 1895-08

**Authors:** 


### EDITORIAL DEPARTMENT.

F. E. DANIEL, M. D., Editor.
S. E. HUDSON, M. D., Managing Editor.
A. J. SMITH. M. D., Galveston, Associate Editor.

EDITORIAL STAFF:

PROF. J. E. THOMPSON, M. D., Texas Medical College, Galveston; Surgery.
PROF. WM. KEILLER, M. D., Texas Medical College, Galveston; Obstetrics and Gynecology.
PROF. DAVID CERNA, M. D., Texas Medical College, Galveston; Therapeutics.
PROF. A. J. SMITH, M. D., Texas Medical College, Galveston; Medicine
DR. R. H. L. BIBB, Saltillo, Mexico; Foreign Correspondent.

Official organ of the West Texas Medical Association, the Houston District Medical Association, the Austin District Medical Society, the Galveston County Medical Society, and several others.

### DR. MERRIMAN ON MANDRAKES.

"Dan'els," said our jolly, fat friend, as he dropped lazily into our easy chair and wheeled himself in front of the electric fan,—"do you ever read the Bible?"

"Cert," said we, too much overcome by the heat of the weather, and the coolness of our visitor acting alternately on our sensibilities, to even finish the sentence; but added mentally, "what do you take us for? Why, doctor?" said I.

"Oh nothing," said the doctor, as he touched the button of our electric "hand-em-around," and helped himself to a twenty-five cent Havana, which we keep on hand only for paying subscribers; "only I was thinking. I have heard the dear good, old people say there is a deal of comfort in the Bible,—and, recently, I was feeling very uncomfortable,—in fact, I was sick, and thought I was going to die; I was scared, I reckon, and I got down the Bible and began to look for comfort, but"—Here the doctor sighed and, shutting his eyes, evidently was deriving comfort from the fragrant weed.

"Didn't you find it?" we inquired.

"Find nothin'. There was mostly 'begittins' and 'begots' in the part I read; and there aint much comfort in that,—to the other fellow,—is there, Dan'els?" and he chuckled a good natured chuckle and went on:—"But I found something there that set me to thinking; Dan'els, what are mandrakes?"

"*Podophyllum peltatum*, commonly called May apple; purgative; plenty of 'em in Mississippi, where you and I came from; 'ask us something hard,'" said we, holding up from proof reading a moment; "why, doctor!"

"You are away off about your podophyllum, Dan'els," said he. "Mandrakes, in Bible days, at least, were something valued very highly, especially by the women folks. Well, I'll tell you the story, and then you'll see what I'm driving at. It is the 30th chapter of Genesis. You know Jacob got stuck on his uncle's little daughter, Rachel—Miss Rachel Laban was her name—and made it all right with her, but the old man was close at a bargain, and he made Jake serve him, 'tending cattle, etc., seven years, before he would agree to the marriage; and then put up a job on him. When the seven years were out, the old man shoved the oldest daughter off on him, Miss Leah. Of course, Jacob kicked, but the old man says, says he—'why, Jake, you soft head, didn't you know 'twas *unlawful* to give the younger daughter in marriage before the older sister had stepped off? go to.' So, Jake took him at his word, and *went* the two, as we will see presently, as it was agreed, if he would serve another seven years, he could have Rachel also, and it came to pass; in seven years more he got the one he was after, and shook Miss Leah. Meantime, however, Leah had a nice little boy named Reuben, and by and by, when Jacob and Rachel were dwelling together in bliss and harmony, and a tent, I suppose,—hope so,—and poor Leah, the cast off, was scuffling for a living, with no one to help her but little Reube,—something happened with mandrakes in it. The Bible records it, and it must be so, and it must be very important; that's what's puzzling me. In the 14th verse, chapter 30, of Genesis, it says: 'And at harvest time, in the wheat fields, Reuben found some mandrakes, and took them to his mother.' Rachel says: 'Give me of thy son's mandrakes.' Leah says: 'Is it no small matter that thou hast taken away my husband, that thou wouldst take away also now my son's mandrakes?' 'Therefore' [there*for*, I suppose] he shall lie with you to-night,' says Rachel. 'Done,' says Leah. So, late that evening, when Leah saw Jacob returning from the field, she ran out to meet him, and says, says she: 'See here; you have to stay with me to-night, for I have *hired you with my son's mandrakes*.'"

"Tut, tut, doctor; hold up there. What are you giving us?" said Bennett, Hudson and "we," all in chorus—while the office boy went into paroxysms of dry grins.

"Fact," says the jolly doctor. "Now, what *are* mandrakes?—what did Rachel want with them so bad that she was willing to lend her husband to a rival woman for just a few of them? As showing they were not the May apple, as you say, which ripens in May,—Reuben found them in harvest time, which must have been in August or September; and as illustrating the value of them, in addition to the fact of hiring out her husband for them,—Leah rated them of value next to her husband. Says she: 'You have taken my husband, now would you also take away my son's mandrakes?' As a man would say: 'You have taken my houses and lands, now will you take also my cattle and horses, and my money?' He wouldn't say: 'You have taken my land and houses, now would you take away also my cat?' If mandrakes had been some trifle, Rachel would have offered some trifle for them, and not, the very first pop, offer that which was dearest to her—usually is to most women—her husband's caresses.

"Now, I've gotten an idea," continued the fat doctor, as he touched the other electric button, and poured himself out a sherry cobbler with ice in it, and a straw, from our other patent electric automatic dumb waiter, which the JOURNAL, like all other truly wealthy people, keeps for the convenience of callers at our sanctum; "I'm of the opinion that it was a 'yarb' of *some* kind—good for female complaints, and that Rachel was the original Lydia E. Pinkham, the concoctor of the celebrated 'vegetable compound.' I can imagine now, with my eyes shut, her advertisement in the *Judah Herald*, or in the *Canaan Evening News*, something like this: 'Mrs. Rachel Jacobs (*nee* Laban) announces to her suffering female friends and the world at large, that she has, at an *enormous sacrifice*, obtained a supply of fresh mandrakes, which she has put into her justly celebrated vegetable compound, and now offers it at a dollar a bottle; warranted to cure all female complaints, etc., etc. Get the genuine.' If not, Dan'els, what *are* mandrakes, and what do you think of the incident recorded in Genesis?"

With that the good doctor unlimbered, and taking his feet off the desk, slowly got up to leave, and looking back over his shoulder, said: "If you find out about those mandrakes, let me know.—I'm going to search the scriptures *again;* there is no telling what I may find. Tata, Dan'els,—so long, Hudson—see you again, Bennett;" and the sunshine went out with the jolly philosopher, the JOURNAL'S standby and friend.

